# Assessing the Long-Term Impact of Treating Hepatitis C Virus (HCV)-Infected People Who Inject Drugs in the UK and the Relationship between Treatment Uptake and Efficacy on Future Infections

**DOI:** 10.1371/journal.pone.0125846

**Published:** 2015-05-04

**Authors:** Hayley Bennett, Phil McEwan, Daniel Sugrue, Anupama Kalsekar, Yong Yuan

**Affiliations:** 1 Health Economics & Outcomes Research Ltd., Cardiff, Wales, United Kingdom; 2 Swansea Centre for Health Economics, Swansea University, Swansea, Wales, United Kingdom; 3 BMS Global Health Economics and Outcomes Research, Princeton, New Jersey, United States of America; Kaohsiung Medical University Hospital, Kaohsiung Medical University, TAIWAN

## Abstract

**Objective:**

The prevalence of the hepatitis C virus (HCV) remains high amongst people who inject drugs (PWID) and accounts for the majority of newly acquired infections. This study aims to quantify the value of treatment amongst PWID with more efficacious treatments and at increased uptake rates, with respect to the avoidance of future infections and subsequent long-term complications of HCV.

**Methods:**

A dynamic HCV transmission and disease progression model was developed, incorporating acute and chronic infection and their long-term complications (decompensated cirrhosis, cancer, liver transplant and mortality), with the potential for HCV transmission to other PWID prior to successful treatment. The model was populated with prevalence and therapy data from a UK setting. Scenarios of current standard of care (SoC) treatment efficacy and uptake were compared to anticipated sustained virologic response (SVR) rates of 90–100% and increased uptake over varied horizons.

**Results:**

SoC led to modest reductions in prevalence; >5% after 200 years. New treatments achieving 90% SVR could reduce prevalence below 5% within 60 years at current uptake rates or within 5 years if all patients are treated. Amongst 4,240 PWID, chronic HCV infections avoided as a result of increasing treatment uptake over the period 2015–2027 ranged from 20–580 and 34–912 with SoC and 90% SVR rates respectively. The reduction in downstream HCV infections due to increasing treatment uptake resulted in an approximate discounted gain of 300 life-years (from avoiding reduced life expectancy from HCV infection) and a gain of 1,700 QALYs (from avoiding the disutility of HCV infection and related complications), with a projected £5.4 million cost saving.

**Conclusion:**

While improved SVR profiles led to reductions in modelled prevalence, increased treatment uptake was the key driver of future infections avoided. Increased treatment among PWID with new more efficacious therapies could significantly change the future dynamics, cost and health burden of HCV-related disease.

## Introduction

The sharing of needles and other injection paraphernalia among injecting drug users is one of the highest risk factors for acquiring the hepatitis C virus (HCV). Globally, approximately 90% of newly acquired HCV infections are attributed to injecting drug use, although this percentage varies by country [1,2]. People who inject drugs (PWID) therefore account for a disproportionately large proportion of newly acquired infections and, despite targeted harm-reduction and education programmes, the incidence and prevalence of HCV amongst PWID remains high.

Interventions aimed at reducing the harms of injecting drug use, and potentially the rate of HCV transmission, have been predicted to reduce future HCV prevalence rates [3–5]. A reduction in HCV incidence in Glasgow has been attributed to the introduction of harm reduction interventions including needle/syringe exchange in the 1980s and 1990s [4]; however, these interventions alone are insufficient to eradicate HCV. In the most recent Needle Exchange Surveillance Initiative (NESI) report, 68% of current and former PWIDs in Glasgow were positive for anti-HCV antibodies [6]. Across Scotland, prevalence rates ranged from 14% to 68% [6]. Figures are similar in other regions of the UK: in 2012, 49%, 33% and 34% of current and former PWID participating in the Unlinked Anonymous Monitoring (UAM) survey in England, Wales and Northern Ireland, respectively, tested positive for antibodies to HCV [7]. These figures demonstrate that behavioural interventions alone are unlikely to alter the prevalence of HCV and highlight the important role that HCV treatment may play in the prevention of future disease transmission.

Clinical guidance in the UK advocates the treatment of HCV in PWID; however, rates of treatment uptake in the clinical setting are low. In 2013, Public Health England estimated that only 3% of HCV positive individuals in the UK are treated annually with therapies approved by the National Institute for Health and Care Excellence (NICE) [8]. The reasons behind this low rate of treatment uptake are numerous and complex. An all-party parliamentary hepatology group (APPHG) report cited a reluctance on the part of clinicians to treat PWID, driven by concerns regarding risk of reinfection, low treatment adherence, high rates of treatment discontinuation, chaotic lifestyles, and high rates of concomitant alcohol abuse and mental health issues [9].

Modelling studies have demonstrated that modest rates of HCV treatment among active PWID could effectively reduce future disease transmission, resulting in a reduction of overall HCV prevalence [10]. The emergence of novel treatments capable of achieving sustained virological response (SVR) rates approaching 100% mean that pharmacological intervention has real potential to alter the future transmission dynamics of the disease [11]. Therefore, the aim of this study was to model the potential value of treating PWID with either current or newer, more effective HCV therapies at various uptake rates in relation to disease transmission, prevalence of chronic HCV and avoidance of long-term complications associated with new infections.

## Materials and Methods

**Fig 1:**
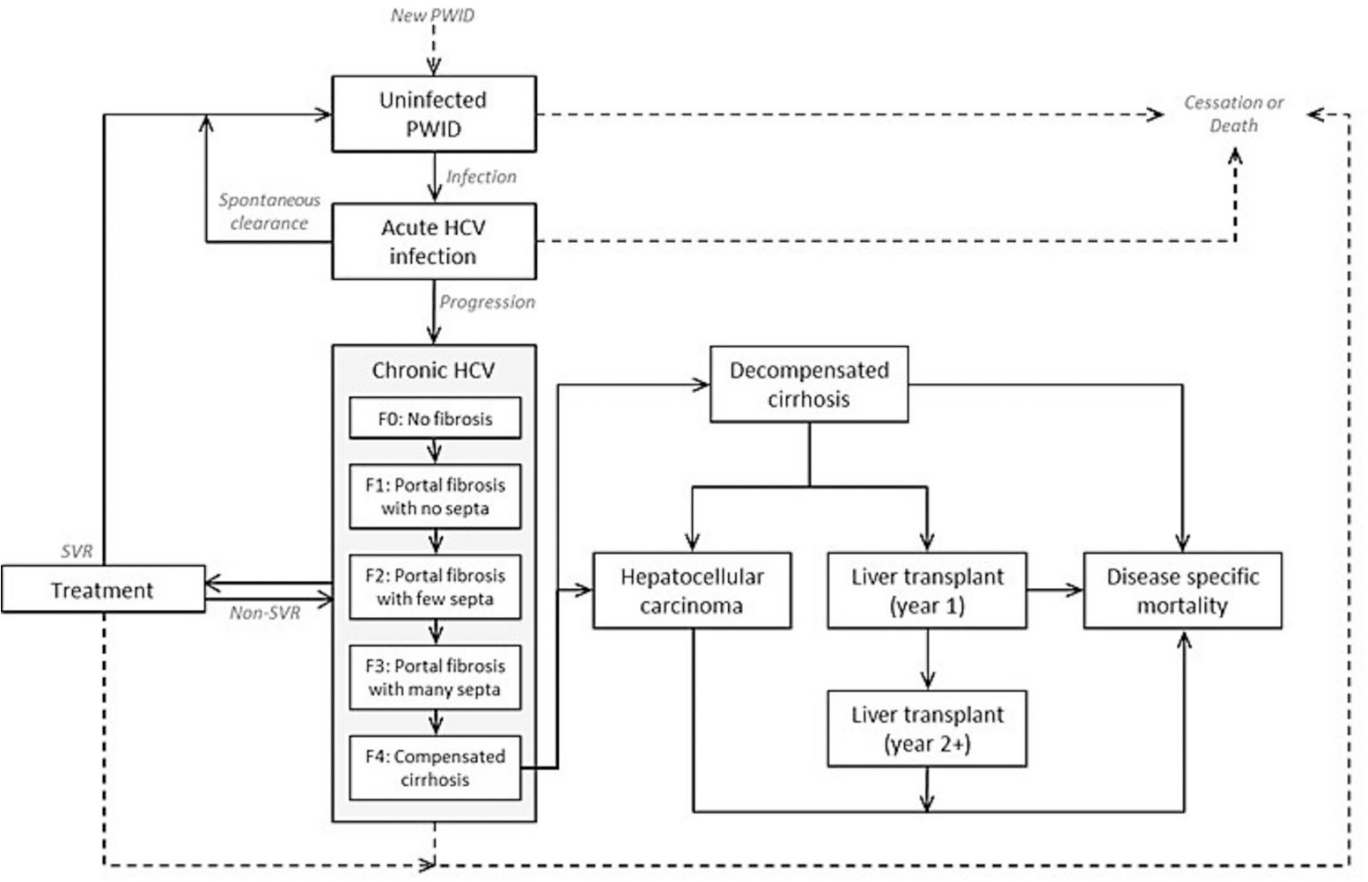
Combined disease transmission and disease progression model schematic. PWID = people who inject drugs; SVR = sustained viral response; HCV = Hepatitis C Virus.

A modelling approach was taken, in which a conventional HCV disease progression model and a disease transmission model were combined. The model structure is shown in Fig 1.

### HCV transmission process

The disease transmission model was developed in Microsoft Excel, based on the published HCV transmission model by Martin *et al*. [2]. The deterministic compartment model represents the acute infection of susceptible individuals who may subsequently clear infection or progress to chronic HCV. If chronically infected individuals are treated, they may achieve SVR and become susceptible once more or fail treatment and remain chronically infected. The PWID population is stratified by transmission risk (low/high) and opiate substitution therapy (OST) status. OST involves medically supervised replacement of illicit opiates with a prescribed substance and it has been proposed that PWID who receive OST have a more predictable lifestyle and could be more easily accessed for receipt of HCV therapy, decreasing transmission risk.

### Data and assumptions

The values for all parameters (Table 1) were derived from data available in Martin *et al*. [2] and are based upon the PWID population in Edinburgh. Key assumptions included that of a fixed-size PWID population; the entry rate of new PWIDs was balanced against the input rates of death and cessation of drug use. The rates of recruitment to OST and transition from low to high risk were balanced against duration of OST and time at high risk, so that the proportions of people at high risk and receiving OST remained constant over time. Following treatment failure, individuals were not retreated. Acute and chronically infected individuals were assumed to be equally infectious.

**Table 1:** Summary of modelling data inputs taken from Martin *et al*. [2].

Parameter	Value	Source
PWID population size	4,240	[12]
Duration of injecting lifetime	11 years	[13,14]
Overall mortality rate	1% per year	[15,16]
Proportion PWID at high risk	33%	[17,18]
Proportion PWID on OST	57%	[6], unpublished ^a^
Duration on OST	8 months	[16]
Duration high risk	14 months	[5,19]
Proportion genotype 1	53%	[20]
Chronic HCV prevalence among PWID in 2012	25%	[6]
Proportion acutely infected spontaneously clearing infection	26%	[21]
Duration acute period	6 months	[22]
Relative risk for HCV while on OST	0.41	[23]
Relative risk for HCV for high risk	3.6	[18,23]
Baseline annual treatment rate	8 per 1,000 PWID	[20], unpublished
**Proportion achieving SVR**		
PEG-IFN+RBV G1	37%	[24]
PEG-IFN+RBV G2/3	67%	[24]
Telaprevir/boceprevir G1	63%	[25,26]
IFN-free DAAs	90%	Estimated; [11,27,28]
**Treatment duration**		
PEG-IFN+RBV (G1)—SVR	48 weeks	[29]
PEG-IFN+RBV (G1)—non SVR	12 weeks	[29]
PEG-IFN+RBV G2/3	24 weeks	[29]
Telaprevir/boceprevir G1	24 weeks	[25,26]
IFN-free DAAs	12 weeks	Estimated; [11]

DAAs: direct-acting antivirals; PEG-IFN: pegylated interferon-alfa; HCV: hepatitis C virus; G: genotype; RBV: ribavirin; SVR: sustained virologic response; PWID: people who inject drugs; OST: opiate substitution therapy

^a^ From 2008/2009 NESI survey excluding those who attended a survey recruitment site for methadone

No treatment was assumed prior to 2002, as there were no NICE-approved therapies available at this time, followed by a linear scale-up in treatment uptake to the baseline annual treatment rate of 8 per 1,000 PWID in 2007. This fixed annual number of treatments was assumed to be constant until 2015. From 2015 to 2017, the modelled treatment rate was further scaled-up linearly, with annual uptake rates of 10, 20, 40, 80, 100, 150, 200 and 250 per 1,000 PWID evaluated. These rates correspond to approximately 4%, 8%, 16%, 32%, 40%, 60%, 80% and 100% of modelled PWIDs chronically infected with HCV in 2012, respectively.

Until 2012, all treatment was assumed to be with a combination of pegylated interferon and ribavirin. From 2012, half of all treated genotype 1 individuals were assumed to receive triple therapy (addition of telaprevir or boceprevir). From 2015, all treated individuals were modelled as receiving new treatments, with varied efficacy in terms of SVR rates.

### Analyses conducted

Analyses focused on evaluating the long-term trends in HCV prevalence (100-year horizon) and the potential impact of introducing newer therapies with improved efficacy and increased treatment uptake on these trends.

The relationship between SVR, treatment uptake and future prevalence of chronic HCV was investigated in more detail over a shorter-term horizon (up to 2027) to establish which component was responsible for the greatest reduction in HCV transmission.

A final analysis aimed to quantify the number of new infections that could be avoided as a result of various treatment strategies over this shorter horizon and estimate the expected long-term HCV complications that may be avoided as a result. This was achieved by linking the disease transmission process to that of a published HCV disease model [30–32]. Chronic HCV is modelled by fibrosis stage (F0–F4), during which patients can be treated. Patients progress from F4 (compensated cirrhosis) to a number of long-term HCV complications: decompensated cirrhosis, hepatocellular carcinoma, liver transplant and death (Fig 1). Lifetime complication rates, costs of complications and impact on quality-adjusted life-years (QALYs) and life-years were derived based on the simulated progression of a 25 year old from fibrosis stage F0 on the METAVIR scoring system over 80 years. These estimates were then applied to the modelled number of new infections avoided between 2015 and 2027, weighted according to the assumption that 3.5% of these new infections would be treated (the approximate proportion of chronic HCV PWIDs treated under the baseline scenario over this period).

## Results

The projected trends of new chronic infections, chronic HCV prevalence and treatment failures were plotted over time and compared against the results of the published HCV model [2]. In line with the findings of Martin *et al*, it was estimated that, to reduce chronic HCV prevalence in Edinburgh by 25%, 50% and 75% at 15 years, the average annual treatment rate would need to be scaled-up to 8.4, 15.4 and 21.8 per 1,000 PWID respectively [2]. Similarly, when comparing the relative prevalence reductions achieved at 15 years by various treatment uptake rates, the results compared well[2]: uptake rates of 8, 10, 20, 40 and 80 per 1,000 PWID lead to reductions of 24%, 31%, 68%, 92% and 93% in prevalence, respectively.

### Long term trends in HCV prevalence

**Fig 2:**
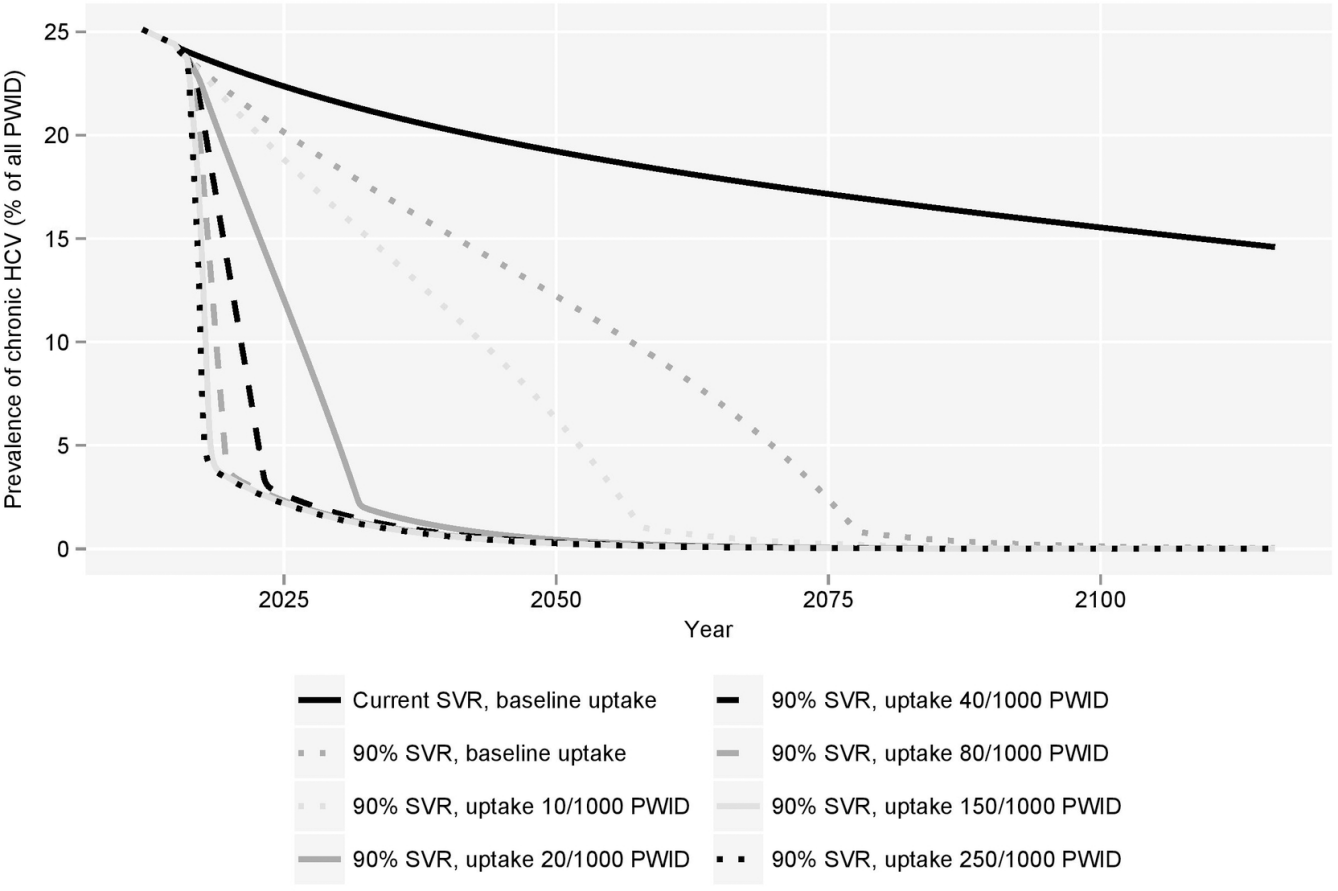
Modelled long-term prevalence of chronic HCV among PWIDs with varied treatment uptake. PWID = people who inject drugs.

Martin *et al* demonstrated that, with current treatment uptake rates, the prevalence of HCV would decline over the next 15 years [2]. However, by projecting the transmission of HCV over a longer time horizon, results from this analysis demonstrated that with the use of current treatments at current uptake rates, the prevalence of chronic HCV would remain in excess of 5% of the total PWID population in 200 years. By contrast, with new treatments estimated to achieve 90% SVR, chronic prevalence would fall below 5% by 2070 and below 1% by 2077 (Fig 2) at the same uptake rate. Over 100 years, the use of such treatments was estimated to prevent more than 5,000 new cases of chronic HCV infection amongst a steady PWID population size of 4,240.

Intuitively, increasing treatment uptake would thus further reduce chronic HCV prevalence. When the modelled treatment uptake rate was increased from a maximum of 8 per 1,000 PWID to 10, 20 or 40 treatments per 1,000 PWID from 2015–2017 onwards, the predicted prevalence of chronic HCV fell to less than 1% of the PWID population by 2058, 2041 and 2036, respectively. If all chronically infected PWID could be treated from 2015–2017 onwards, a prevalence of less than 1% could be achieved within 20 years.

### Relationship between SVR and treatment uptake

**Fig 3:**
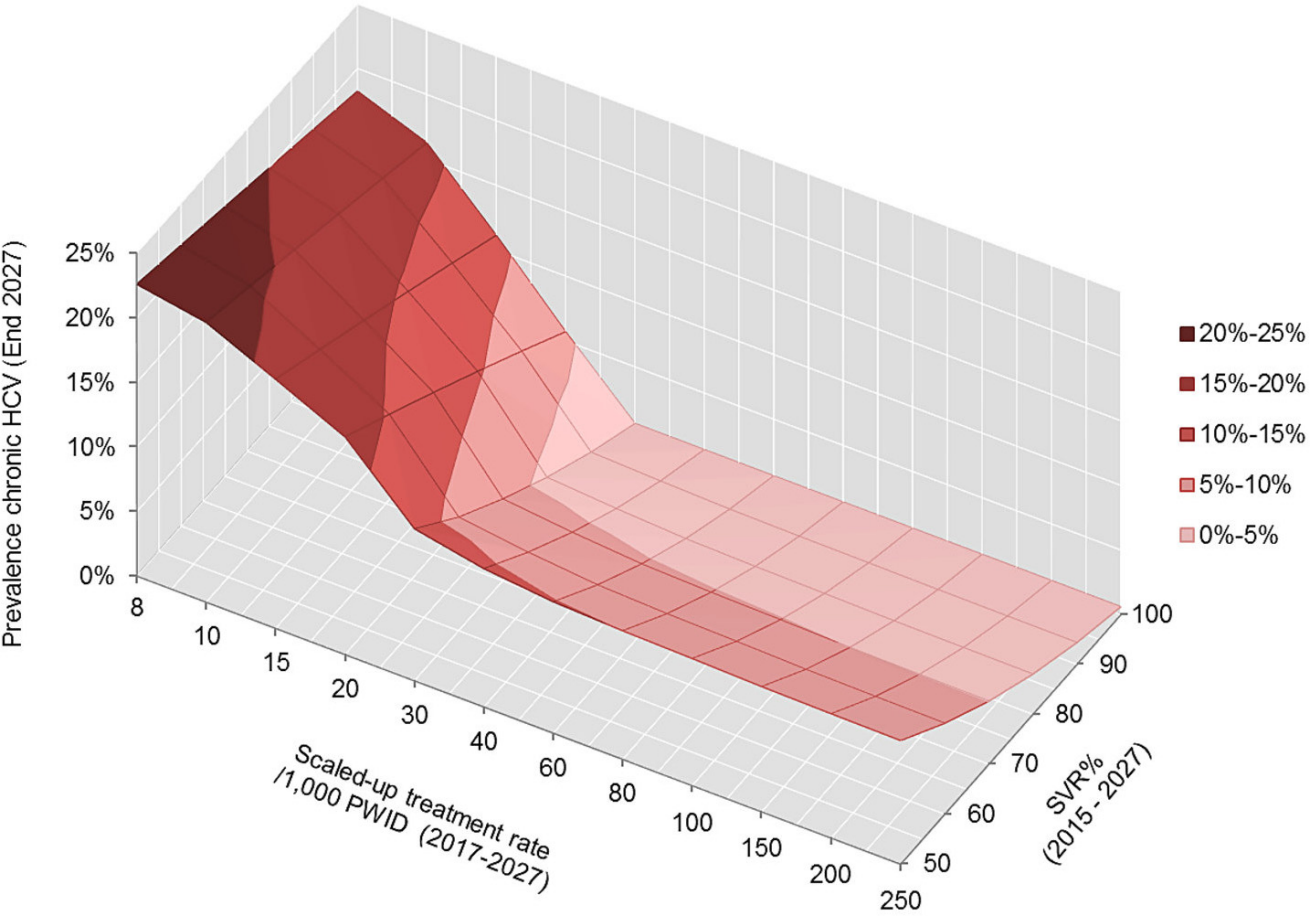
Prevalence of chronic HCV amongst PWIDs at the end of 2027 as a function of SVR rate achieved with new therapies and scaled-up treatment rate. PWID = people who inject drugs.

The model was used to predict prevalence of chronic HCV at the end of 2027 following the introduction of new treatments (2015–2017) and 10 years of treatment (2017–2027) at the scaled-up treatment rate (Fig 3). Results illustrate that, while an improved SVR profile can lead to a reduction in HCV prevalence, it is the treatment uptake rate that is the key driver. For example, at the current uptake rate of 8 per 1,000 PWID, an increase in SVR from 50% to 100% was estimated to achieve an absolute reduction in chronic HCV prevalence of less than 5%, compared to an 11.2% reduction achieved by increasing treatment uptake from 8 to 20 per 1,000 PWID at current SVR rates.

### New chronic infections avoided and downstream consequences

Over the period 2015–2027, results suggest that significant numbers of new infections could be avoided. At current SVR rates, between 20 and 580 new chronic HCV infections were estimated to be avoided amongst a steady population size of 4,240 PWID as a result of increasing treatment uptake rates to between 10 and 250 per 1,000 PWID. At an SVR rate of 90%, this could increase to between 34 and 912 new infections avoided.

**Fig 4:**
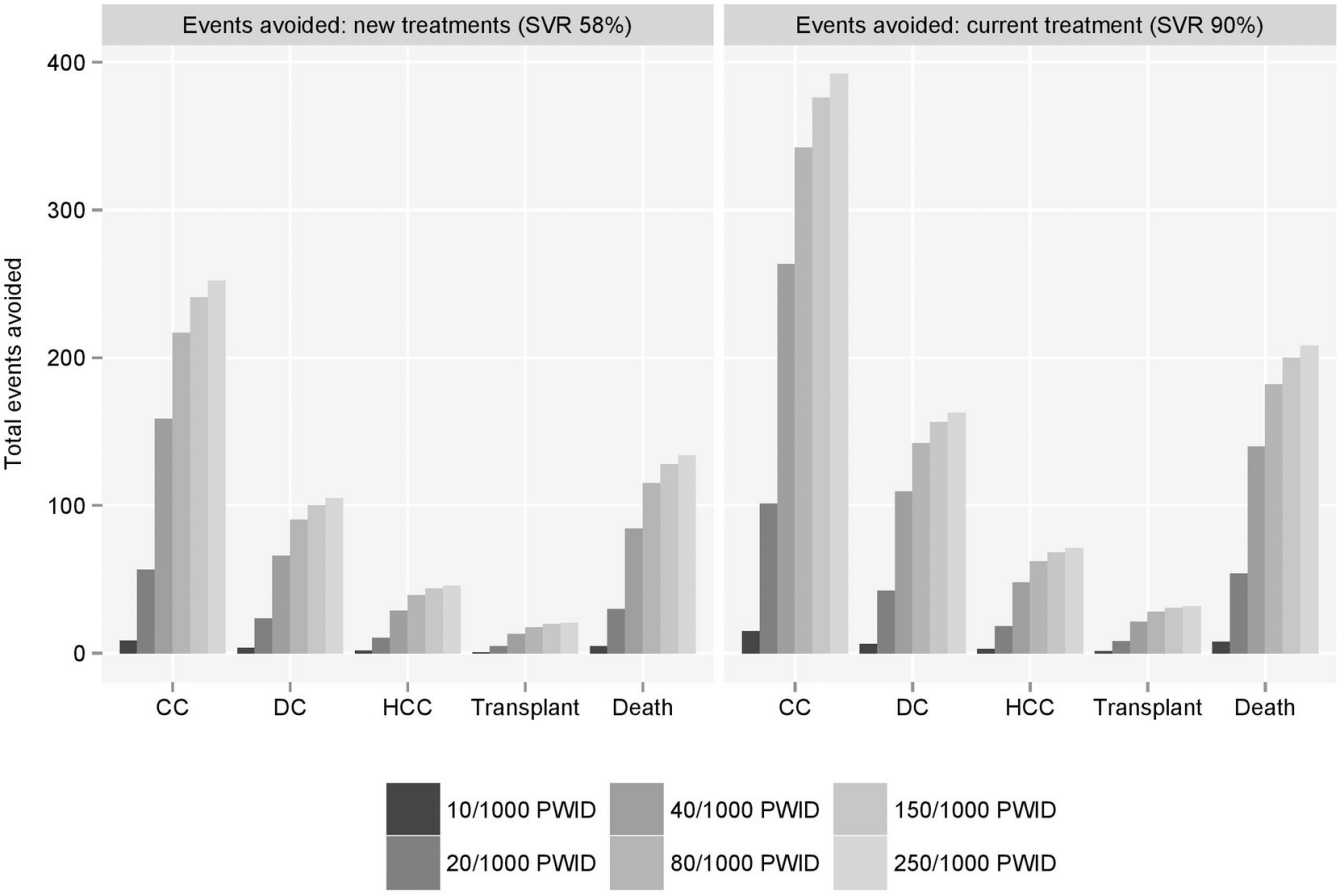
Future complications avoided as a result of new chronic HCV infections avoided compared to baseline treatment uptake (8/1000 PWID). PWID = people who inject drugs; SVR = sustained viral response.

**Fig 5:**
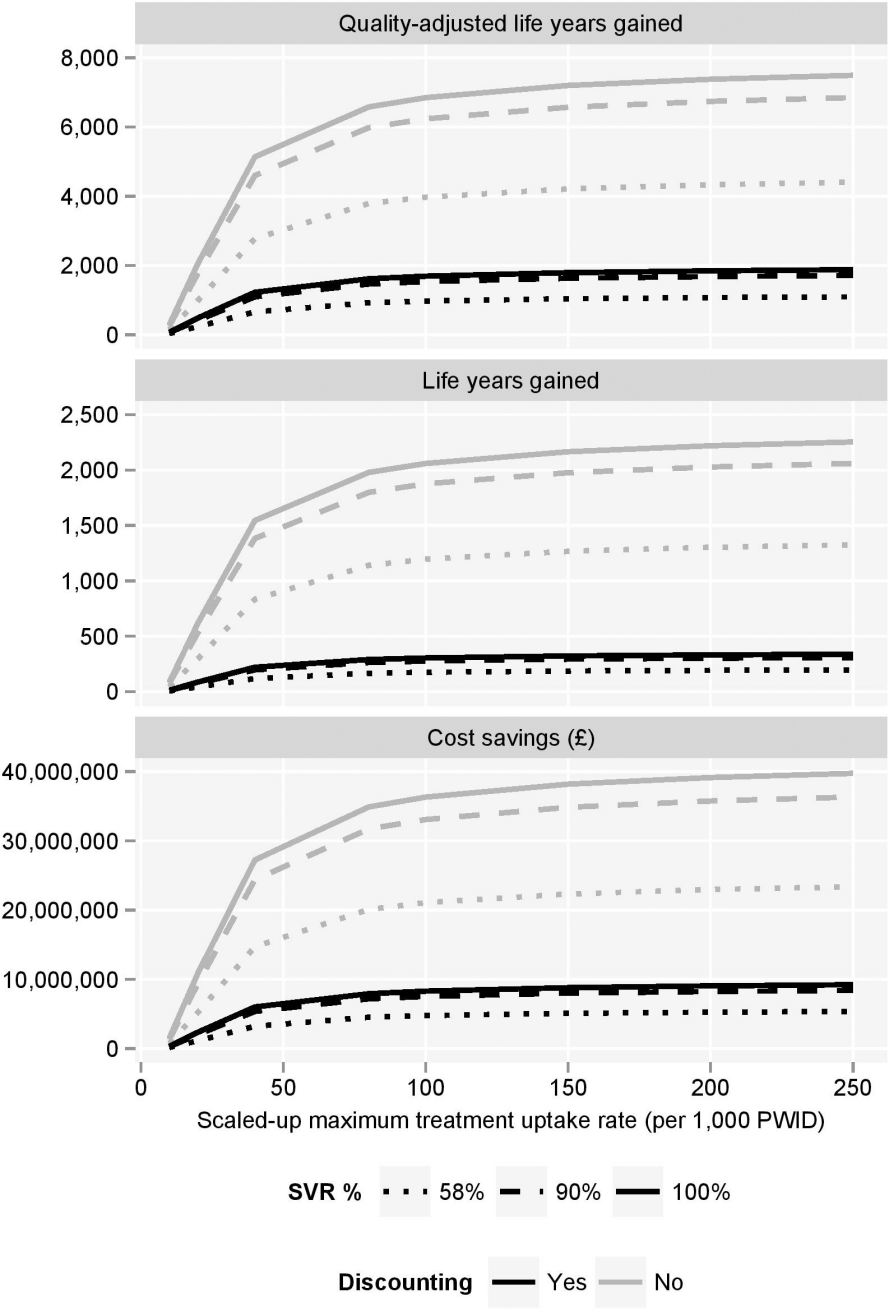
Cost savings, quality-adjusted life year gains and life year gains made as a result of the avoiding future complications of new chronic HCV infections. PWID = people who inject drugs.

Without treatment, approximately 44% of these new infections were estimated to lead to compensated cirrhosis, 18.4% to decompensated cirrhosis, 8.1% to hepatocellular carcinoma, 3.6% to liver transplant and 23.6% to death due to liver-related causes. With treatments that can achieve SVR in 90% of patients, these proportions reduce to 11.1%, 4.6%, 2.0%, 0.9% and 5.9%, respectively. Consequently, the number of long-term, HCV-related complications that could be avoided through prevention of new infections is non-trivial (Fig 4). Furthermore, the future downstream costs, healthcare resource utilisation and implications for survival and quality of life are significant (Fig 5).

Through increased uptake (up to 250 per 1,000 PWID) of current treatment in the modelled population (4,240 PWID), up to 46 cancers, 21 liver transplants and 134 liver-related deaths could be prevented amongst new chronic infections alone. The avoidance of these modelled complications relates to an estimated absolute saving of approximately £23.4 million, or £5.4 million when costs are discounted at 3.5% per annum. At 90% SVR, estimated numbers increased to 71 cancers, 32 transplants and 208 liver-related deaths avoided at a total saving of £36.3 million, or discounted saving of £8.4 million (Fig 5). At the highest treatment uptake rate, 90% SVR was estimated to provide absolute gains of approximately 2,000 life-years and 6,800 QALYs, and discounted gains of approximately 300 life-years and 1,700 QALYs, compared to the baseline uptake rate.

## Discussion

This study explored the potential impact of HCV treatment with newer, more efficacious therapies on future disease transmission and the avoidance of long term complications associated with new infections in PWID. The results presented support previous studies that considered HCV treatment as prevention in demonstrating that HCV treatment could reduce HCV transmission and prevalence among PWID [2,10,33–37]. At current levels of HCV treatment, with current SVR rates, modest reductions in HCV chronic prevalence were projected, consistent with previous studies; however, the predicted prevalence among the PWID population was still estimated to exceed 5% in 200 years. Increasing treatment uptake could dramatically reduce prevalence in a much shorter time scale: treating 20 infections per year per 1,000 PWID with a 25% HCV prevalence is estimated to result in a 68% reduction in HCV prevalence over 15 years, in line with previous estimates [2].

Among a steady population of less than 5,000 PWID, treating up to 250 per 1,000 PWID was estimated to prevent up to 46 cancers, 21 liver transplants and 134 liver-related deaths through reduced disease transmission and the avoidance of new chronic infections. The potential avoidance of such complications related to an estimated absolute saving of approximately £23.4 million, or £5.4 million when costs are discounted. Although a strategy of treating all chronically infected individuals would initially require a large increase in the annual number of treatments administered and associated healthcare costs, the resultant fall in chronic HCV prevalence over time would lead to fewer treatments required and considerable long-term cost offsets due to the avoidance of new infections and complications. Theoretically, if all chronically infected PWID could be treated from 2015–2017 onwards, a prevalence of less than 1% could be achieved within 20 years.

These projections are based on a theoretical mathematical model, which have inherent limitations and incorporate a number of assumptions. Analysis focused solely on the PWID population and did not address the spread of HCV through other routes, such as sexual contact, contaminated blood products or blood transfusion or mother to child transmission. However, PWID are acknowledged to be the key drivers of HCV transmission [1,38]; indeed, recommendations have been made to focus public health efforts on treating this population in the aim of significantly reducing or even eradicating disease [38].

The model assumes a stable injecting population size. HCV disease prevalence and transmission rates were obtained from published epidemiological data on PWID in the UK, and hence these results are UK-specific. The 2012 prevalence rate of 25% used in this study corresponded to the prevalence of HCV in NHS Lothian, in line with the Edinburgh parameterisation presented by Martin *et al. [2]*. However, the prevalence of HCV within PWID populations of the UK varies widely by geographical location. Presented results may under- or over-estimate benefits in populations where HCV prevalence rates differ significantly.

As the aim of this study was to explore the potential value of newer, more efficacious therapies and scale-up of antiviral treatment, the model incorporated current levels of OST. The impact of increasing coverage or targeting of drug treatment or harm reduction interventions, which may also contribute to reducing HCV transmission among PWID, were not explored; though even modest reductions have been shown to require long-term sustained coverage [5]. Eligibility criteria for these programmes or the potential impact of characteristics of drug use such as frequency, duration, sharing networks or drug type were also not considered.

Accessing PWID will play a significant part in the success of increasing treatment uptake in practice. This study did not address the complexities involved in treatment upscale or the practical implications of identifying infected individuals and treating more difficult cases, issues likely to become more pronounced if prevalence can be reduced and the number of patients treated represents a larger proportion of the infected population. While OST programmes may offer a platform for the delivery of treatment in the community, additional healthcare infrastructure, resources and training may be required as part of further interventions such as screening or those addressing the stigma associated with testing and treatment.

In Australia, Canada, Europe and the United States, programmes designed to address barriers to care among PWID have achieved yearly HCV treatment rates of 40–80 per 1,000 PWID with pegylated interferon-alfa and ribavirin dual therapy [39–42]. Improved efficacy and tolerability of emerging therapies, which require shorter durations of treatment, may further contribute to the successful delivery of treatment among PWID. If these rates could be achieved in the UK, substantial cost savings and quality of life improvements could be made as a result of reducing the number currently infected and the future incidence of chronic infections and HCV-related complications.

The effectiveness of new HCV therapies was assumed to be in the range of 90–100% SVR, based on phase 2 and 3 studies [43–48]. Outcomes of therapy in PWID are unknown, but systematic reviews report similar response rates among PWID and non-PWID for current standard of care [36,49]. This analysis is limited to those PWID with HCV infection only; HIV/HCV co-infected individuals have not been modelled.

By linking disease transmission and HCV disease progression modelling, the importance of evaluating the significant health and cost implications associated with future infections as part of a disease transmission analysis has been highlighted. Conversely, the implications of future disease transmission should be considered when modelling the consequences of treatment as part of HCV disease progression modelling. The health economic evaluation of new antiviral therapies, particularly those appropriate for difficult to treat patients at high risk of transmission, should account for the preventive impact of treatment on future transmission.

## Conclusion

The benefits of HCV treatment, in terms of quality of life, survival, healthcare resource use and associated costs, extend beyond the treated individual, particularly in populations at higher risk of disease transmission. Increased treatment rates among PWID with more efficacious treatments has the potential to significantly change the future dynamics and burden of HCV-related disease, which is of critical public health importance given the lack of alternative and effective HCV prevention strategies. Whether the scale-up required to eradicate disease is both affordable and achievable remains an important challenge for both clinicians and public health policy makers to address.
